# Serum uric acid levels during leprosy reaction episodes

**DOI:** 10.7717/peerj.1799

**Published:** 2016-03-14

**Authors:** Yvelise T. Morato-Conceicao, Eduardo R. Alves-Junior, Talita A. Arruda, Jose C. Lopes, Cor J.F. Fontes

**Affiliations:** 1 Department of Internal Medicine, Faculty of Medicine, Julio Muller University Hospital, Universidade Federal de Mato Grosso, Cuiaba MT, Brazil; 2 Department of Biomedicine, Centro Universitário UNIVAG, Varzea Grande MT, Brazil; 3 Department of Internal Medicine, Julio Muller University Hospital, Universidade Federal de Mato Grosso, Cuiaba MT, Brazil

**Keywords:** Leprosy reactions, Uric acid, Oxidative stress

## Abstract

**Background.** Leprosy reactions are acute inflammatory episodes that occur mainly in the multibacillary forms of the disease. The reactions are classified as type 1 (reverse reaction) or type 2 (erythema nodosum leprosum). Leprosy-associated oxidative stress has been widely demonstrated. Several recent studies have shown uric acid (UA) to have antioxidative effects under pathologic conditions. The objective of this study was to assess serum levels of UA in patients with leprosy reactions, with the aim of monitoring their levels before and after treatment, compared with levels in leprosy patients without reactions.

**Methods.** The study included patients aged 18–69 years assisted at a leprosy treatment reference center in the Central Region of Brazil. Patients who were pregnant; were using immunosuppressant drugs or immunobiologicals; or had an autoimmune disease, human immunodeficiency virus infection, acquired immune deficiency syndrome, or tuberculosis were excluded. Upon recruitment, all individuals were clinically assessed for skin lesions and neural or systemic impairment. Some patients had already completed treatment for leprosy, while others were still undergoing treatment or had initiated treatment after being admitted. The treatment of the reactional episode was started only after the initial evaluation. Laboratory assessments were performed upon admission (baseline) and at approximately 30 and 60 days (time points 1 and 2, respectively).

**Results.** A total of 123 leprosy patients were recruited between June 2012 and June 2015; among them, 56, 42, and 25 presented with type 1, type 2, and no reactions, respectively. Serum UA levels were significantly reduced in patients with type 2 leprosy reactions compared with patients in the control group and remained lower in the two subsequent assessments, after initiation of anti-reaction treatments, with similar values to those recorded before the treatment.

**Discussion.** The decreased serum UA levels in patients with type 2 leprosy reactions might be due to the consumption of UA to neutralize the enhanced production of oxygen- and nitrogen-reactive species that occurs during type 2 reactions. The maintenance of the reduced levels in the follow-up assessments may indicate persistence of oxidative stress in the initial post-treatment stages, despite improved clinical conditions. The results of this study suggest that serum UA may play an antioxidative role during type 2 leprosy reactions.

## Introduction

Despite worldwide efforts to control leprosy through early detection and adequate multidrug therapy (MDT) treatment, this disease remains a significant health problem in countries such as India, Brazil, and Indonesia, which showed the highest disease incidence in 2013 (World Health Organization, 2014). With the aim of leprosy elimination, the World Health Organization (WHO) recently put forward actions intended to decrease disability or sequelae, specifically targeting leprosy reactions (World Health Organization, 2014). Leprosy reactions are acute inflammatory episodes resulting from exacerbated host immune responses, which may occur during the natural progression of the disease as well as during and after treatment. The reactions are related to the bacillary load and are classified as type 1 (reverse reaction) or type 2 (erythema nodosum leprosum) (Scollard et al., 2006). Between 30% and 50% of leprosy patients may experience leprosy reactions (Scollard et al., 2015).

Erythema nodosum leprosum involves acute inflammation of the organs and tissues invaded by the bacilli, and the dermal vasculature and subcutaneous tissue are severely compromised (Nogueira et al., 2000). The typical clinical sign of this reaction is the sudden appearance a large number of painful erythematous nodules throughout the body. In the most serious cases, they may evolve to form vesicles, blisters, or necrotic phenomena and the patients also present systemic symptoms (Kahawita & Lockwood, 2008). The recurrence of type 2 reactions is frequent and may persist for years (Scollard et al., 2006). Laboratory observations comprise increased acute-phase proteins including C-reactive protein (CRP), *α*1-antitrypsin (Foss, 2003; Kahawita & Lockwood, 2008), *α*1-acid glycoprotein (AGP) (Gupta, Shankernarayan & Dharmalingam, 2010), and *γ*-globulins (Foss, 2003). Treatment of type 2 reactions consists of administration of prednisone or thalidomide. Thalidomide is the drug of choice in Brazil (Brazilian Ministry of Health, 2014).

Type 1 reactions are caused by activation of the Th1 immune response to *Mycobacterium leprae* antigens. Clinically, increased inflammation of pre-existing skin lesions is observed and, different from type 2 reactions, intense neuritis occurs, which may lead to both sensory and motor neuron impairment; systemic symptoms are rare, and visceral impairment does not occur. Treatment of type 1 reactions in Brazil includes glucocorticoids (Nery et al., 2013).

*M. leprae* is an obligatory intracellular bacterium that replicates slowly (12–14 days) inside macrophages and Schwann cells (Suzuki et al., 2012). Activated macrophages destroy phagocytosed bacteria through the action of microbicide molecules inside phagolysosomes. These include proteolytic enzymes such as elastase and cathepsin G, reactive oxygen species, and reactive nitrogen intermediates (Abbas, Lichtman & Pillai, 2008). These molecules act synergistically and may cause extracellular tissue damage through changes in the structure or function of proteins, membrane lipids, and nucleic acids (Kohen & Nyska, 2002). Antioxidant substances are produced to control the levels of reactive oxygen species (hydroxyl radical, superoxide anion, and hydrogen peroxide, among others) and reactive nitrogen derivatives (mainly nitric oxide). Those antioxidant substances may be either enzymatic, such as glutathione peroxidase, catalase, and superoxide dismutase (SOD), or nonenzymatic, such as vitamin E (tocopherol), vitamin C (ascorbic acid), reduced glutathione, uric acid (UA), *β*-carotene, transferrin, and ceruloplasmin (Dröge, 2002; Kohen & Nyska, 2002).

Disequilibrium between the generation and neutralization of reactive species is called oxidative stress. Oxidative stress may occur in conditions that involve chronic inflammation such as systemic hypertension, type 2 diabetes mellitus, and heart failure (Kohen & Nyska, 2002); cancer (Gaman et al., 2014); degenerative neurologic diseases (Dröge, 2002); autoimmune diseases (Dröge, 2002; Pérez et al., 2012); or infectious diseases such as malaria (Clark & Hunt, 1983; Becker et al., 2004), human immunodeficiency virus infection (Aukrust et al., 2003), dengue (Olagnier et al., 2014), and Chagas disease (Zacks, 2005).

Oxidative stress has been described in type 1 reactions (Chhabra et al., 2015) and multibacillary (MB) forms of leprosy (Bhadwat & Borade, 2000; Reddy et al., 2003; Prasad, Admath & Admath, 2007; Jyothi et al., 2008; Abdel-Hafez, Mohamed & Abd-Elghany, 2010; Schalcher et al., 2013; Swathi & Tagore, 2015) and may be worsened by the patient’s nutritional status and by the MDT itself (Schalcher et al., 2014).

UA is the final product of purine metabolism and is produced by the oxidation of hypoxanthine and xanthine by xanthine oxidase (Ames et al., 1981). UA belongs to a damage-associated molecular pattern group of molecules recognized by the immune system as a danger signal (Ghaemi-Oskouie & Shi, 2011; Crane & Mongiardo, 2014). In most countries, UA serum levels between 2.6 and 6.0 mg/dL in women before menopause, and between 3.5 and 7.2 mg/dL in women after menopause and men, are considered normal (Desideri et al., 2014).

In parallel with its inflammatory activity, UA itself has potent antioxidant properties, reacting with the peroxyl radical and nitrogen dioxide and chelating transition metals; it is responsible for up to 60% of the antioxidant capacity of human blood, and its plasma concentration is higher than that of other antioxidants, such as vitamins C and E (Ames et al., 1981). Both high and low UA serum levels are associated with increased cardiovascular mortality and loss of renal function (Bergamini et al., 2009; Meotti et al., 2011; Kanda et al., 2015). A review article by Fang et al. (2013) reports studies in which low UA serum levels were observed in patients with neurodegenerative diseases, suggesting that the antioxidant action of UA may play a role in preventing neurodegeneration.

The objective of this study was to assess UA serum levels in patients with or without leprosy reactions and to monitor their levels before and after reaction treatment.

## Methods

The study included 123 patients treated at University Hospital Júlio Muller of the Mato Grosso Federal University (Cuiabá, Mato Grosso, Brazil) between June 2012 and June 2015, by either spontaneous decision or referral. Individuals younger than 18 or older than 69 years of age, pregnant women, carriers of autoimmune or neoplastic diseases, patients using immunosuppressive drugs or immunobiologicals, and patients with HIV/AIDS, tuberculosis, or another mycobacteriosis were excluded. The study was approved by the Julio Muller University Hospital Ethics Committee for Human Research (number 19502). All study participants signed informed consent forms.

All patients underwent clinical skin and neurologic examinations. Blood was collected by peripheral venous puncture on three occasions: upon admission (baseline) and approximately 30 and 60 days later (time points 1 and 2, respectively). Patients were classified according to the operational classification from the WHO adopted by the Brazilian Health Ministry in 2001 (Brasil, 2001), which rates cases with up to five skin lesions and/or a single compromised nerve trunk as PB and cases with more than five skin lesions and/or more than one compromised nerve trunk as MB. Each clinical diagnosis was complemented by a slit-skin smear examination and calculation of the bacillary index (four to five collection sites). Patients with up to five skin lesions but with positive microscopy findings were classified as MB. All patients were treated with MDT according to the WHO guidelines (World Health Organization, 2014). Upon admission, some patients had already completed the MDT, while others were still undergoing treatment or had not begun treatment for leprosy. The diagnosis of reactions and neuritis was made by physicians with experience in their diagnosis and treatment and was confirmed by a second specialist physician. The treatment of the reactional episode was started only after admission into the study and after the baseline blood collection. All patients with type 1 reactions were given prednisone orally, 40–80 mg/day, for 4 weeks and then prednisone was tapered off. Type 2 reactions were treated with thalidomide (100–400 mg/day, based on clinical status) except when contraindicated; prednisone was associated in the cases of concomitant neuritis and/or necrotizing erythema nodosum (Scollard et al., 2006; Brazilian Ministry of Health, 2014).

Routine laboratory tests were performed, including hemograms and assessments of lipid profile, erythrocyte sedimentation rate, hepatic enzyme, blood glucose, UA, lactic dehydrogenase, urea, and creatinine levels. CRP and AGP, which constitute classical acute-phase markers, were quantified and used as an indirect measure of oxidative stress. Automated quantification of serum UA levels using an enzymatic method was performed using a CT600i (Thermo Fisher Scientific, Schwerte, Germany) according to the manufacturer’s instructions. The glomerular filtration rate (eGFR) was estimated by the CKD-EPI (Chronic Kidney Disease Epidemiology Collaboration equation) method (Matsushita et al., 2012).

Participants were divided into three groups: those without a reaction (controls), those with type 1 reactions, and those with type 2 reactions. For data analysis, data from patients with type 1 reactions and patients with neuritis only were pooled. Statistical analysis was performed using the statistics package Stata^®^ (version 12) and the freely available program Epidata^®^ Analysis (version 2.1.1). The Student’s *t*-test was used to compare means of serum UA levels in the groups with and without reaction, with Bonferroni correction for multiple comparisons when applied. However, when the variances of UA levels were not homogeneous between groups, they were compared using the nonparametric Mann–Whitney or Kruskal–Wallis tests. Following that analysis, the variables that presented statistically significant associations were included in a stratified analysis model that controlled for the following risk factors for oxidative stress: age, smoking, alcoholism, hypertension, hyperlipidemia, and diabetes. The nonparametric paired Wilcoxon test was used to assess differences in serum levels of UA, CRP, and AGP in post-treatment evaluations. An *α* error of 0.05 was used for all statistical analyses.

## Results

This study included 123 patients with leprosy who spontaneously sought treatment at the reference center (25.2%), who were referred for treatment of leprosy reactions (69,1%), or who were summoned for screening (5.7%) owing to being household contacts of patients with leprosy. Nine patients were classified as having PB and 114 as having MB leprosy. A slit-skin smear examination was performed for 84.6% of patients and was positive in 83.6% of cases. Most patients were men (74.0%) between 18 and 69 years of age (mean: 41.4 years). The most frequent comorbidities were systemic hypertension (8.9%) and smoking (19.5%). Seven patients presented hyperlipidemia: six in the group with type 1 reaction and one in the group with type 2 reaction. Sixty-two percent of the study participants did not present any comorbidity. Three patients had reduced eGFR (<60 mL/min/1.73m^2^) in the baseline, two of which had type 2 reaction and evolved with normalization of the eGFR in subsequent evaluations; the other patient had type 1 reaction and did not attend the follow-up visits.

In the initial assessment, 25 patients (20.3%) did not present leprosy reactions, 10 (8.1%) presented only neuritis, 46 (37.4%) presented type 1 reactions, and 42 (34.2%) presented type 2 reactions. Neuritis was possible, but not universal, among patients in the last two groups with leprosy reactions (Table 1). MDT was initiated upon study admission in 37 patients: 13 without reaction, 20 with type 1 reactions, and four with type 2 reactions. Forty-one patients (32.3%) had already completed leprosy treatment (28 with type 2 reactions and 13 with type 1 reactions), and 45 were still undergoing MDT: 12 without a reaction, 23 with type 1 reactions, and 10 with type 2 reactions. The disability assessment examination performed at the start of treatment showed some degree of functional impairment in 53 individuals (43.1%).

**Table 1:** Demographic and clinical data of the 123 patients included in the study.

Characteristic		*n*	%
Sex	Male	91	74.0
	Female	32	26.0
Race	White	52	42.3
	Brown	55	44.7
	Black	16	13.0
Age (years)	18 ⊣ 30	29	23.6
	30 ⊣ 40	32	26.0
	40 ⊣ 50	27	22.0
	>50	35	28.4
	Mean (SD): 41.4 (12.6)		
Operational classification	Paucibacillary	9	7.3
	Multibacillary	114	92.7
Microscopy	Positive	87	70.7
	Negative	17	13.8
	Not performed	19	15.5
Bacilloscopy index^*^	0 ⊢ 3	46	51.7
	3 ⊢ 6	43	48.3
	Mean (SD): 2.63 (1.85)		
Leprosy reaction	Type 1	56	46.3
	Type 2	42	34.7
	No reaction	25	19.0
Comorbidities^**^	Type 2 diabetes mellitus	2	1.6
	Hypertension	11	8.9
	Hyperlipidemia	7	5.7
	Smoking	24	19.5
	Alcoholism	7	5.7
	Others	7	5.7
Reaction treatment	Thalidomide	23	23.5
	Prednisone	58	59.2
	Thalidomide + prednisone	17	17.3

**Notes.**

* Not available for 15 patients with positive microscopy.

** Some patients presented more than one comorbidity.

To treat the reactional episode, prednisone was used in all cases of type 1 reaction and in two young female patients with type 2 reaction. Thalidomide was the drug of choice in most of the type 2 reactions; prednisone was associated in the cases of concomitant neuritis and/or necrotizing erythema nodosum (Table 1).

The mean (SD) serum UA levels were compared between patients with and without known risk factors for oxidative stress. Significantly higher UA serum levels (*p* = 0.006) were observed in patients with hyperlipidemia. No statistically significant associations were found for the remaining risk factors (Table 2).

**Table 2:** Univariate analysis of serum uric acid levels before treatment of 114 patients, according to risk factors for oxidative stress.

Risk factors		Uric acid (mg/dL)		p^*^
		Mean	SD	
Age (years)	18 ⊣ 30	4.04	0.94	0.149^**^
	30 ⊣ 40	4.63	1.16	
	40 ⊣ 50	4.95	1.63	
	>50	4.54	1.86	
Smoking	Yes	4.69	1.26	0.311
	No	4.50	1.53	
Alcoholism	Yes	4.66	1.54	0.415
	No	4.53	1.48	
Hyperlipidemia	Yes	6.88	2.48	0.006
	No	4.43	1.33	
Hypertension	Yes	4.80	1.86	0.825
	No	4.51	1.45	
Type 2 diabetes mellitus	Yes	4.15	1.20	0.658
	No	4.54	1.48	

**Notes.**

* Mann–Whitney test.

** Kruskal–Wallis test.

The present study showed that serum UA levels were significantly reduced in patients with type 2 leprosy reactions compared with levels in patients in the control group (*p* = 0.023). This difference was still significant after the Bonferroni correction for multiple comparisons (*p* = 0.046). The serum levels of UA were also different between patients with type 2 and type 1 leprosy reactions (*p* = 0.033). However, this difference was not maintained (*p* = 0.112) after excluding patients with hyperlipidemia, which predominated in the group with type 1 reaction (Table 3).

**Table 3:** Serum levels of uric acid at initial assessment (before treatment) of patients with and without leprosy reactions.

Patient group	*n*	Uric acid (mg/dL)		p^*^
		Mean	SD	
Type 1 reaction	75	4.75	1.66	0.857
Control		4.82	1.43	
Type 2 reaction	64	4.08	1.14	0.023^a^
Control		4.82	1.43	
Type 2 reaction	89	4.08	1.14	0.033^b^
Type 1 reaction		4.75	1.66	

**Notes.**

* Student’s *t*-test.

a *p* = 0.046 after Bonferroni correction.

b *p* = 0.112 after exclusion of hyperlipidemic patients (*n* = 84; the uric acid of one patient was not evaluated).

**Figure 1:**
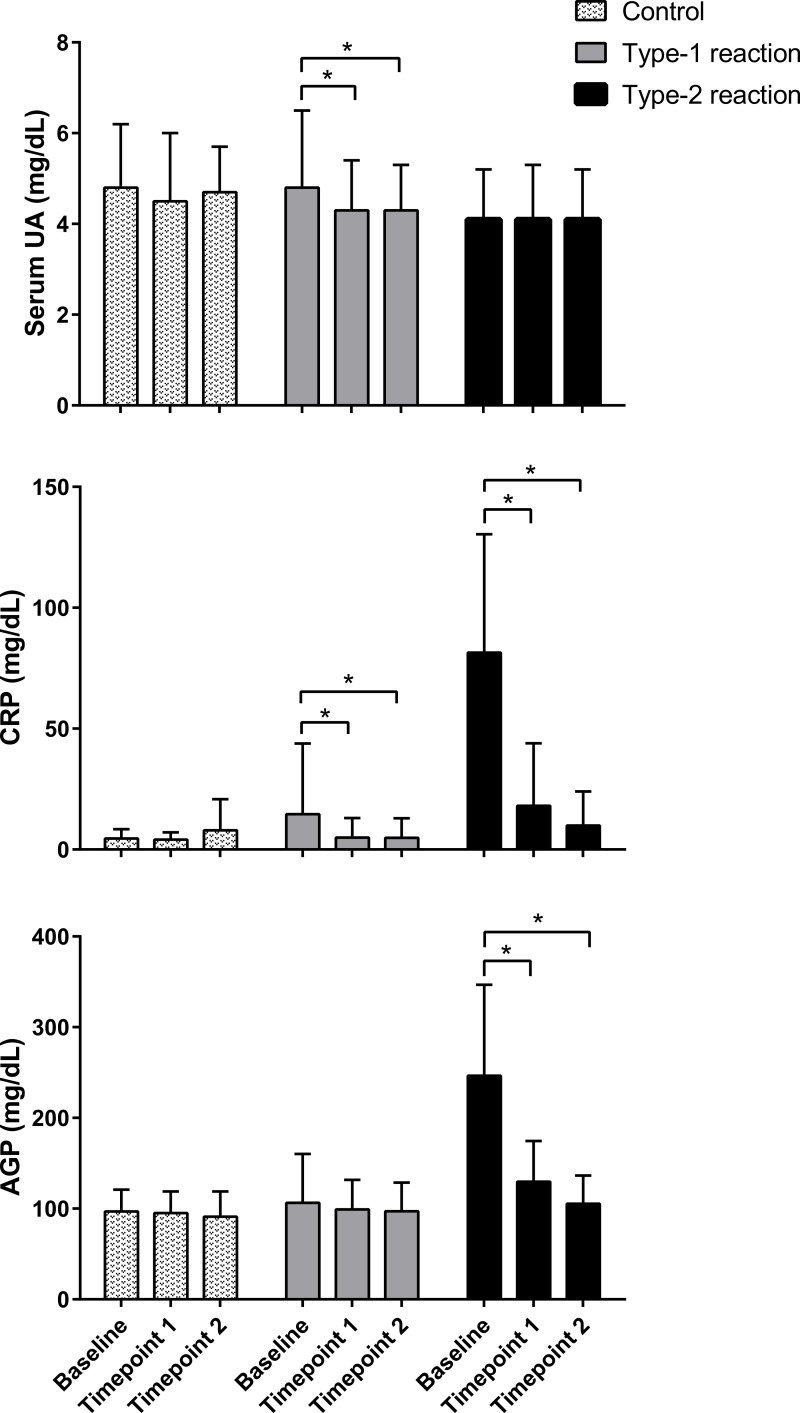
Follow-up evaluation of serum levels of uric acid, C-reactive protein, and *α*1-acid glycoprotein in patients with type 1 and type 2 leprosy reaction and in control patients. The means ± standard deviation were calculated from patients upon admission (baseline) and at approximately 30 and 60 days (time points 1 and 2, respectively). *n* = 114, 117 and 115 at baseline; 103, 104, and 106 at time point 1; and 85, 83, and 87 at time point 2, respectively, for serum UA (uric acid), CRP (C reactive protein), and AGP (*α*1-acid glycoprotein). * *p* < 0.05.

Serum UA, CRP, and AGP were measured, respectively, in 114, 117, and 115 patients at baseline; 103, 104, and 106 patients at time point 1; and 85, 83, and 87 patients at time point 2. Losses were mainly due to study withdrawal as well as technical problems with the blood sample during laboratory procedures. Comparison of serum levels of UA, CRP, and AGP in follow-up assessments showed that the levels of these molecules remained within the normal range in patients without reactions (control) and remained stable in all assessments. At baseline, patients with type 1 reactions had normal serum levels of UA and AGP, whereas CRP levels were slightly elevated. In post-treatment assessments, serum levels of UA (*p* = 0.003 and *p* = 0.002) and CRP (*p* = 0.016 and *p* = 0.004) were significantly lower after 30–60 days (time point 1) and 60–90 days (time point 2) of prednisone treatment, respectively, compared with pre-treatment levels. Serum levels of AGP remained within the normal range and did not change significantly during post-treatment follow-up assessments (Fig. 1).

However, among patients with type 2 reactions, serum levels of CRP (*p* < 0.0001) and AGP (*p* < 0.0001) were high at baseline and time point 1 and returned to the normal range at time point 2. This behavior was not observed for UA; in fact, the serum levels of UA remained low in both assessments after treatment for type 2 reactions, and their values were similar (*p* = 0.694 at time point 1 and *p* = 0.649 at time point 2) to baseline serum levels (Fig. 1).

## Discussion

In the present study, the mean serum UA levels were lower in leprosy patients with type 2 reactions than in patients without reactions. However, no statistically significant differences were observed between patients with type 1 reactions and patients without reactions. To the best of our knowledge, this is the first report of changes in UA levels as a result of leprosy reactions. The significantly lower UA levels in patients with type 2 leprosy reactions suggest increased consumption of UA to neutralize the increased production of reactive species that occurs during type 2 reactions (Foss, 1997). Conversely, the lack of association between serum UA and type 1 reactions may be explained by a lower production of reactive species, which may be too low to distinguish patients with type 1 reactions from those without reactions. Another hypothesis would be the higher frequency of hyperlipidemic patients in the group with type 1 reaction, which may be associated with hyperuricemia (Desideri et al., 2014; Wei et al., 2015) and which could minimize the association between serum UA level and type 1 reaction.

The observation that the mean serum UA levels remained relatively consistent before and after treatment in patients with type 2 reactions may suggest the persistence of oxidative stress, despite clinical improvement, and decreased serum levels of acute-phase proteins such as CRP and AGP. Chhabra et al. (2015) and Prabhakar et al. (2013) also described the persistence of oxidative stress markers after clinical resolution in leprosy patients treated for type 1 reactions and after completing MDT, respectively.

A significant decrease in serum UA levels, attributed to its possible antioxidant effect, was described in patients with pemphigus vulgaris, particularly in those with mucosal impairment (Yousefi et al., 2011), lichen planus (Chakraborti et al., 2014), and neurodegenerative diseases (Keizman et al., 2009; Fang et al., 2013). The hypothesis that serum UA is consumed during type 2 reactions is consistent with previous findings regarding the profile of enzymatic antioxidants in patients with polar forms of leprosy; i.e., the antioxidant level decreases gradually from the tuberculoid to the virchowian pole. The authors suggested that a high bacillary load might lead to increased production of free radicals, which would lead to consumption of antioxidant molecules (Jyothi et al., 2008).

In general, low serum UA levels may result from its decreased synthesis or increased consumption or excretion. Primary hypouricemia may arise from xanthine oxidase deficiency or a defect in the renal tubular transport of UA, with decreased UA reabsorption leading to very low serum levels of this molecule (≤2.0 mg/dL) (Martín & García-Nieto, 2011; Iso & Kurabayashi, 2015). This situation may occur in diseases that cause proximal tubular impairment and uricosuria, such as Fanconi’s syndrome, Wilson’s disease, inadequate secretion of antidiuretic hormone syndrome, and myeloma (Martín & García-Nieto, 2011). However, none of our patients presented symptoms or signs compatible with these diseases. Some medications may also lead to decreased serum UA levels through various mechanisms, including decreased UA production (allopurinol), inhibition of tubular reabsorption (probenecid, ascorbic acid, losartan, sulfinpyrazone, and benzbromarone), or increased excretion (polyethylene glycol-uricase) (McDonagh et al., 2014). Losartan stands out among drugs with reported effects on serum UA levels. This was the only drug used by three patients in this study, one in the control group and two in the group of patients with type 1 leprosy reactions.

In most cases, a correlation was observed between decreased serum UA levels after treatment in patients with type 1 reactions and the duration of the use of the maximum dose of prednisone (40–80 mg/day), when patients presented clinical improvement and apparent control of reaction-associated inflammation, as shown by decreased CRP serum levels. Based on these considerations, increased or stabilized UA levels would be expected. This observation could be explained by increased renal excretion of UA due to the action of the glucocorticoid. A significant decrease in serum UA levels was observed in patients with heart failure and hyperuricemia who received 30–60 mg/day prednisone for 5–28 days (Liu et al., 2013; Meng et al., 2015). This decrease was explained by the enhanced renal response to endogenous natriuretic peptides (NPs), through the upregulation of the expression of NP receptor A in the intramedullary renal collecting duct, with the consequent induction of potent diuresis, in rats with chronic excessive cardiac insufficiency (Liu et al., 2011). NPs are part of a family of hormones usually produced in the vasculature, myocardium, kidneys, adrenal glands, brain, and lungs. They play an important role in the maintenance of cardiovascular homeostasis (Simões e Silva, Pinheiro & Santos, 2008). Three genetically distinct but structurally related NPs are known: atrial NP, type B NP, and type C NP. ANP seems to be responsible for the control of normal cardiorenal activity, whereas increased secretion of the other two hormones occurs only under pathological conditions (Volpe, 2014).

Some limitations of this study should be highlighted, such as the low number of patients included and the short follow-up time of the patients, which is required to understand the actual dynamics of serum UA levels. A selection bias was possible, as the patients were recruited from a reference center where demands concerning leprosy reactions are more frequent and there are relatively fewer control patients without reactions. Not measuring the urinary concentration of UA of the patients with the objective of removing hypersecretory patients also represents another limitation of this study. Information on water intake, purine contents in diet, and exercise levels, which was not obtained from the patients, may also represent a limitation. However, we believe that these factors did not influence the results of this study, since the patients were homogenous in terms of cultural background and socioeconomic level. In addition, during the reaction episodes, physical activity tends to be very restricted due to neural pain and systemic symptoms.

The results of this study suggest that UA might be consumed in order to neutralize the increased production of oxygen and nitrogen reactive species during type 2 reactions in patients with leprosy. In addition, the maintenance of serum UA levels at values similar to those observed before treatment might indicate that oxidative stress persists in the initial post-treatment phases, despite clinical improvement and decreased serum levels of acute-phase markers (CRP and AGP). These findings suggest that serum UA may play an antioxidative role during type 2 leprosy reactions. Additional studies with a greater number of patients are needed to confirm this hypothesis.

## Supplemental Information

10.7717/peerj.1799/supp-1 Supplemental Information 1 Dataset

10.7717/peerj.1799/supp-2 Supplemental Information 2 Dataset encoding

## Additional Information and Declarations

### Data Availability

The following information was supplied regarding data availability: The raw data has been supplied as Supplemental Information.
