# Supplementary material for: Serum uric acid levels during leprosy reaction episodes
Source: PeerJ. 2016 Mar 14;4:e1799. doi: 10.7717/peerj.1799 (PMC4793330; doi:10.7717/peerj.1799)
Supplement: Supplemental Information 2 [file peerj-04-1799-s002.docx]

**DATABASE:** uricacidleprosy.xlsx

**ENCODING USED**

clinical_presentation:

1: Without reaction

2: Type 1 reaction

3: Type 2 reaction

who_classification

1: paucibacillary

2: multibacillary

bacillary_index:

9: positive, but bacillary index unknown

sex:

0: female

1: male

race:

1: white

2: brown

3: black

age_group:

1: 18 ┤30 years

2: 30 ┤40 years

3: 40 ┤50 years

4: >50 years

type1_reaction:

0: absent

1: present

type2_reaction:

0: absent

1: present

smoking:

0: no

1: yes

alcoholism:

0: no

1: yes

hypertension:

0: no

1: yes

diabetes:

0: no

1: yes

hyperlipidemia:

0: no

1: yes

**α-1-acid glycoprotein (alfa1gp)**

alfa1gp1: baseline

alfa1gp2: timepoint 1

alfa1gp3: timepoint 2

**C-reactive protein (crp)**

crp1: baseline

crp2: timepoint 1

crp3: timepoint 2

**Serum uric acid (ua)**

ua1: baseline

ua2: timepoint 1

ua3: timepoint 2
